# Supplementary material for: Examining Canadian youth’s engagement with food companies via digital media
Source: PLOS Digit Health. 2025 Dec 30;4(12):e0001167. doi: 10.1371/journal.pdig.0001167 (PMC12753059; doi:10.1371/journal.pdig.0001167)
Supplement: S1 Table — (PDF) [file pdig.0001167.s002.pdf]

SUPPLEMENTARY MATERIAL

TITLE: Examining Canadian youth's engagement with food companies via digital media

AUTHORS: Laura Vergeer[1], Meghan Pritchard[1], Carolina Soto[1], Elise Pauzé[2], Ashley Amson[2], Dana Lee Olstad[3], Monique Potvin Kent[1]

AFFILIATIONS:

[1]School of Epidemiology and Public Health, Faculty of Medicine, University of Ottawa, Ottawa, Ontario, Canada

[2]Interdisciplinary School of Health Sciences, University of Ottawa, Ottawa, Ontario, Canada

[3]Department of Community Health Sciences, Cumming School of Medicine, University of Calgary, Calgary, Alberta, Canada

CORRESPONDING AUTHOR: Monique Potvin Kent (monique.potvinkent@uottawa.ca)

Supplementary Table 1. The number and percentage of participants in the analytic sample who identified as each racial/ethnic group and as having each level of income adequacy (n=1162).

Race/ethnicity	n (%)
Race/ethnicity	
White	877 (75.5)
Racial/ethnic minority group	
Black	40 (3.4)
East Asian	66 (5.7)
Indigenous	5 (0.4)
Latin	28 (2.4)
Middle Eastern	26 (2.2)
South Asian	51 (4.4)
Southeast Asian	15 (1.3)
Other or mixed	54 (4.6)
Income adequacy[a]	
Very low	77 (6.6)
Low	315 (27.1)
Medium	450 (38.7)
High	237 (20.4)
Very high	83 (7.1)

[a]Assessed based on the question: "Thinking about your total monthly income, how difficult or easy is it for you to make ends meet?". Response options included: "very difficult" (i.e., very low income adequacy); "difficult" (low income adequacy); "neither easy nor difficult" (medium income adequacy); "easy" (high income adequacy); and "very easy" (very high income adequacy).
