# Supplementary material for: Examining Canadian youth’s engagement with food companies via digital media
Source: PLOS Digit Health. 2025 Dec 30;4(12):e0001167. doi: 10.1371/journal.pdig.0001167 (PMC12753059; doi:10.1371/journal.pdig.0001167)
Supplement: Attachment — Submitted filename: Responses to reviewers-Sep 19 2025.docx [file pdig.0001167.s003.docx]

**Manuscript ID:** PDIG-D-25-00443

**Title:** Examining Canadian youth’s engagement with food companies via digital media

**Authors:** Laura Vergeer, Meghan Pritchard, Carolina Soto, Elise Pauzé, Ashley Amson, Dana Lee Olstad, Monique Potvin Kent

**NOTE:** The page and line numbers below refer to the manuscript version with Track Changes.

**Journal Requirements:**

**COMMENT:** 1. Please send a completed 'Competing Interests' statement, including any COIs declared by your co-authors. If you have no competing interests to declare, please state "The authors have declared that no competing interests exist". Otherwise please declare all competing interests beginning with the statement "I have read the journal's policy and the authors of this manuscript have the following competing interests:"

**RESPONSE:** We have added that statement to the beginning of our ‘Competing Interests’ section (page 26, lines 578-579).

**COMMENT:** 2. Please ensure that your Ethics Statement is available in its entirety at the beginning of your Methods section, under a subheading 'Ethics Statement'. It must include:

1) The name(s) of the Institutional Review Board(s) or Ethics Committee(s)

2) The approval number(s), or a statement that approval was granted by the named board(s)

3) (for human participants/donors) - A statement that formal consent was obtained (must state whether verbal/written) OR the reason consent was not obtained (e.g. anonymity).

NOTE: If child participants, the statement must declare that formal consent was obtained from the parent/guardian.

**RESPONSE:** This section has been added (Methods section, page 7, lines 155-158).

**COMMENT:** 3. Please amend your detailed Financial Disclosure statement. This is published with the article. It must therefore be completed in full sentences and contain the exact wording you wish to be published.

i. State the initials, alongside each funding source, of each author to receive each grant.

**RESPONSE:** We have edited our Financial Disclosure statement (page 26, lines 572-576).

**COMMENT:** 4. In the online submission form, you indicated that “Data generated or analyzed during this study are available from the corresponding author upon reasonable request.”.

All PLOS journals now require all data underlying the findings described in their manuscript to be freely available to other researchers, either 
1. In a public repository, 
2. Within the manuscript itself, or 
3. Uploaded as supplementary information.

This policy applies to all data except where public deposition would breach compliance with the protocol approved by your research ethics board. If your data cannot be made publicly available for ethical or legal reasons (e.g., public availability would compromise patient privacy), please explain your reasons by return email and your exemption request will be escalated to the editor for approval. Your exemption request will be handled independently and will not hold up the peer review process, but will need to be resolved should your manuscript be accepted for publication. One of the Editorial team will then be in touch if there are any issues.

If the reviewer comments include a recommendation to cite specific previously published works, please review and evaluate these publications to determine whether they are relevant and should be cited. There is no requirement to cite these works unless the editor has indicated otherwise.

**RESPONSE:** We have uploaded the data used in this study as supplementary information. Our Data Availability statement has also been updated (page 26, lines 569-570).

**Additional Editor Comments (if provided):**

[Note: HTML markup is below. Please do not edit.]

**Reviewers' Comments:**

Reviewer's Responses to Questions

**Comments to the Author**

1. Does this manuscript meet PLOS Digital Health’s [publication criteria](https://track.editorialmanager.com/CL0/https:%2F%2Fjournals.plos.org%2Fdigitalhealth%2Fs%2Fjournal-information%23loc-criteria-for-publication/1/010f0198a310556b-12f00c6d-06c9-4a2f-a53e-1f38dbabb2a3-000000/3cQIascThaqowW58rGnimGrrK6bRypfb2AcgjCCgeH4=224)? Is the manuscript technically sound, and do the data support the conclusions? The manuscript must describe methodologically and ethically rigorous research with conclusions that are appropriately drawn based on the data presented.

Reviewer #1: Yes

Reviewer #2: No

Reviewer #3: Partly

2. Has the statistical analysis been performed appropriately and rigorously?

Reviewer #1: Yes

Reviewer #2: Yes

Reviewer #3: Yes

3. Have the authors made all data underlying the findings in their manuscript fully available (please refer to the Data Availability Statement at the start of the manuscript PDF file)?

The [PLOS Data policy](https://track.editorialmanager.com/CL0/https:%2F%2Fjournals.plos.org%2Fdigitalhealth%2Fs%2Fdata-availability/1/010f0198a310556b-12f00c6d-06c9-4a2f-a53e-1f38dbabb2a3-000000/nW7tRSyRpwD3Q5hVmjq4wEb0QyXHrR36fp_rCPzjfOQ=224) requires authors to make all data underlying the findings described in their manuscript fully available without restriction, with rare exception. The data should be provided as part of the manuscript or its supporting information, or deposited to a public repository. For example, in addition to summary statistics, the data points behind means, medians and variance measures should be available. If there are restrictions on publicly sharing data—e.g. participant privacy or use of data from a third party—those must be specified.

Reviewer #1: Yes

Reviewer #2: No

Reviewer #3: Yes

4. Is the manuscript presented in an intelligible fashion and written in standard English?

PLOS Digital Health does not copyedit accepted manuscripts, so the language in submitted articles must be clear, correct, and unambiguous. Any typographical or grammatical errors should be corrected at revision, so please note any specific errors here.

Reviewer #1: Yes

Reviewer #2: Yes

Reviewer #3: Yes

5. Review Comments to the Author

Please use the space provided to explain your answers to the questions above. You may also include additional comments for the author, including concerns about dual publication, research ethics, or publication ethics. (Please upload your review as an attachment if it exceeds 20,000 characters)

Reviewer #1:

**COMMENT:** The manuscript offers a valuable and timely contribution by examining Canadian youths’ engagement with food companies via digital media, a topic that is increasingly relevant in the context of rising childhood obesity and unregulated digital food marketing. The study is commendable for its large, stratified sample, well-defined variables, and clear statistical approach, and it importantly highlights sociodemographic disparities in digital marketing exposure.

**RESPONSE:** We thank the reviewer for taking the time to review our manuscript. We have addressed their comments below and in the revised manuscript.

**COMMENT:** However, several improvements could enhance the clarity and impact of the work. First, the operational definition of "engagement" (i.e., liking/sharing/following food companies and having apps on smartphones) should be emphasized more explicitly throughout the abstract, methods, and results to distinguish passive exposure from active interaction.

**RESPONSE:** We added a brief definition of “engagement” to the Abstract (page 2, lines 26-27), the Author Summary (page 3, lines 63-65) and the beginning of the Discussion (page 17, lines 395-396) so that it is clear to the reader what is meant by this term. In the Methods and Results sections, the different outcomes (i.e.: liking, sharing or following food and restaurant companies on social media; having apps for food/restaurant companies on smartphone; having food delivery service apps) are clearly defined and we make clear distinctions between them. Therefore, we do not feel there is a need or a fitting place to reemphasize the operational definition of “engagement” in these sections.

**COMMENT:** Second, while the study reports the presence of food-related apps, no data were collected on the frequency, purpose, or context of use—an important limitation that should be discussed in more depth, along with how these behaviors may differ across age or income groups.

**RESPONSE:** We have expanded on this limitation in the Discussion (pages 23-24, lines 537-541).

**COMMENT**: Additionally, the representativeness of the sample requires more nuanced discussion; given the overrepresentation of White and higher-income participants, potential biases in the findings should be critically evaluated.

**RESPONSE:** This limitation is noted in the Discussion section and we have expanded on it slightly (page 24, lines 544-545) but we’re unsure what else can be said here. While our sample cannot be considered nationally representative, we do think it is important to consider that the proportion of White and high income adequacy household participants n our sample is consistent with national population statistics (Discussion section, page 24, lines 545-547).

**COMMENT:** The manuscript also reports no significant gender differences in overall engagement, but interesting differences appear in brand preferences and motivations (e.g., girls were more likely to follow food companies because the food looks tasty). These gendered behavioral nuances warrant deeper exploration, possibly supported by qualitative insights or future research.

**RESPONSE:** We have noted this as a possible direction for future research (Discussion section, page 19, lines 427-429). No qualitative data was collected in this study.

**COMMENT:** Moreover, the cross-sectional design limits causal interpretations; the authors could strengthen the discussion by acknowledging how longitudinal or experimental designs might better assess directionality and health impacts of engagement.

**RESPONSE:** We have added this limitation and direction for future research to the Discussion section (page 23, lines 535-537).

**COMMENT:** From a policy perspective, the manuscript does an excellent job advocating for tighter digital media regulations, but it could further elaborate on how schools, parents, and media literacy programs might also serve as protective factors against persuasive marketing.

**RESPONSE:** We have added discussion about other possible strategies such as parental involvement and strengthening youth’s media literacy (Discussion section, pages 22-23, lines 511-521).

**COMMENT:** Finally, while data are available on request, the authors are encouraged to consider depositing de-identified datasets in an open-access repository to align with best practices in open science.

**RESPONSE:** The dataset has been included as a Supporting Information file. Our Data Availability statement has also been updated (page 26, lines 569-570).

**COMMENT:** Overall, with minor revisions focused on improving clarity, contextual depth, and discussion of limitations, this manuscript will make a strong contribution to the literature on digital food marketing and public health.

**RESPONSE:** We thank the reviewer for these positive comments on our manuscript.

Reviewer #2:

**COMMENT:** Thank you for the opportunity to review this manuscript. In this work, the authors describe the results of an online survey that was administered to children and adolescents in Canada. The goal of the survey was to assess the digital exposure of Canadian children and adolescents to food marketing, via apps and social media.
The study tackles an interesting issue in public health. Overall, the data has been collected rigorously, and the statistical analysis has been performed adequately.

**RESPONSE:** We thank the reviewer for their positive feedback on our manuscript.

**COMMENT:** However, I have three significant concerns about the manuscript that, in my opinion, raise serious questions about its suitability for publication. First, the authors drive some strong conclusions from their analyses which are not fully supported by the results. In particular, it is unclear from the analysis if the type of engagement with food marketing apps or social media recorded from survey participants may lead to unhealthy or risky behaviors. The manuscript does not report whether children or adolescents exposed to food marketing were more likely to eat unhealthy food or not. Therefore, while it might be interesting to characterize the engagement with food marketing, it is hard to derive conclusions, especially about policy. The evidence to support the need for policy interventions focused on food company apps and food delivery apps is not sufficiently clear from the data.

**RESPONSE:** Examining whether engagement with food companies via digital media is associated with increased marketing exposure and consumption of unhealthy foods was beyond the scope of the present study. However, we have cited other studies in our Discussion that support these associations (page 18, lines 407-410; page 21, lines 482-492). The discussion of our findings does not imply that we found engagement with food marketing to be predictive of unhealthy food consumption. Rather, our results indicate that many youths are engaging with food companies via digital media and this is a public health problem that warrants government policy intervention, given that previous research has shown that youth are exposed to various food marketing strategies on restaurant and food delivery service apps (Bennett et al. Obes Rev. 2024; Gupta et al. Public Health Nutr. 2025) and that youth who engage with food brands online are more likely to consume unhealthy foods (Baldwin et al. Public Health Nutr. 2018).

**COMMENT:** Second, it appears that the authors have already published the results related to my first concern. Upon review, I found that the survey data used in this study have been analyzed previously—on at least two occasions—with findings published in other journals (Refs. 25 and 26). Notably, Ref. 26 presents the very relationship that is absent from the current manuscript and that would be essential to strengthen its conclusions. The repeated use of the same dataset across (at least) three different manuscripts raises concerns about the originality of each contribution and, in turn, diminishes the scientific value of the current work. Furthermore, Ref. 16, authored by the same researchers, is still under review and not publicly accessible. However, its title suggests that it may closely overlap with the current manuscript in terms of content. Is Ref. 16 also based on the same survey data? If so, this raises a serious concern.

**RESPONSE:** The reviewer is correct in that data from this survey have been published in three manuscripts:

1. Vergeer et al. Youth exposure to unhealthy digital food marketing in relation to race/ethnicity and income adequacy in Canada. BMC Nutr. 2025 Aug 11;11(1):163.
2. Vergeer et al. Examining differences in exposure to digital marketing of unhealthy foods reported by Canadian children and adolescents in two policy environments. BMC Nutr. 2025 Feb 7;11(1):32.
3. Vergeer et al. The relationship between youth's exposure to unhealthy digital food marketing and their dietary intake in Canada. Appl Physiol Nutr Metab. 2024 Dec 1;49(12):1678-1691.

However, the objectives of these studies and the outcome variables examined within them are unrelated to the present study. All the studies stem from a cohort study using a survey covering many topics related to digital marketing and that the survey was designed to allow the assessment of multiple research questions. Notably, none of the previously published studies examined any outcomes related to food delivery service and restaurant apps. We therefore see no reason for concern and do not agree that the scientific value of the current study is diminished by the previously published studies.

**COMMENT:** Finally, PLOS Digital Health requires all papers to adhere to the highest standards of open science. Unfortunately, the data used in this study is not available. Given that the data can be readily pseudo-anonymized and deposited in a public repository, this omission is difficult to justify. Open data is particularly crucial in this case, as it appears that multiple publications have already been derived from the same dataset. Without access to the underlying data, the results of this study—as well as those of the related publications—cannot be independently verified or reproduced.

**RESPONSE:** The dataset has been included as a Supporting Information file. Our Data Availability statement has also been updated (page 26, lines 569-570).

Reviewer #3: Overall Assessment
**COMMENT:** The manuscript addresses an important public health concern by examining youth exposure to digital marketing in Canada. However, several methodological and interpretative limitations reduce the strength and generalizability of the findings. Below, I provide major and minor recommendations to improve the rigor, representativeness, and methodological alignment with current computing trends.

**RESPONSE:** We thank the reviewer for their feedback on our manuscript.
________________________________________
Major Comments

**COMMENT:** 1. Sample Representativeness and Geographic Scope
o The dataset consists of 1,162 observations collected only from Ontario and Quebec. While these provinces are significant in population size, limiting the sample to two provinces raises concerns about geographic and demographic representativeness for the entire Canada, as shown in the title.
o Recommendation: Expand data collection to include additional areas to ensure the findings reflect diverse socio-economic, cultural, and regional contexts.

**RESPONSE:** We agree that the representativeness of our sample is limited by the fact that our sample was recruited from only Ontario and Quebec. This is noted as a limitation in the Discussion section of our manuscript (page 23, lines 530-533). The reason these two provinces were selected was because of their very different policy environments concerning food marketing, given food marketing to children in Quebec is regulated under the Consumer Protection Act, while Ontario is limited to voluntary self-regulatory industry initiatives concerning food marketing to children. We have future studies planned that will recruit from across Canada.

**COMMENT:** 2. Sample Size and Statistical Power
o The relatively small sample size (n = 1,162) may limit statistical power and the ability to detect meaningful associations. It also underutilizes the potential of modern computational resources, which can be acquired from larger datasets.
o Recommendation: Increase the sample size in future studies to enhance statistical reliability.

**RESPONSE:** We will strive to increase our sample size in future studies. The survey was administered to 1200 respondents based on a sample size calculation, permitting us to estimate mean responses to within +/- 16% of one SD within each sex/region/age stratum and provide 80% power to detect a small-to-moderate effect (Cohen’s d) of 0.33 between sex/region/age strata.

**COMMENT:** 3. Policy Recommendations and Rationale
o The author’s primary policy recommendation is to restrict youth exposure to digital marketing. While this is a potentially effective intervention, the manuscript does not sufficiently justify why this measure is prioritized over, or in place of, other possible strategies.
o Recommendation: Provide a balanced discussion of alternative or complementary policy measures, such as promoting healthier product reformulation, implementing targeted educational campaigns, or incentivizing corporate responsibility initiatives. This would strengthen the policy relevance and applicability of the findings.

**RESPONSE:** We have added discussion of alternative or complementary policy measures (Discussion section, pages 22-23, lines 511-521).

**COMMENT:** 4. Methodological Approach and Model Selection
o The study employs traditional logistic regression, which is appropriate for binary classification tasks. However, relying solely on this approach may limit the model’s ability to capture complex, non-linear relationships in the data.
o Recommendation: Consider incorporating state-of-the-art machine learning models, such as TabNet with explainability features, in parallel with logistic regression. This would (a) improve predictive accuracy, (b) provide richer insights into feature importance, and (c) align the analytical approach with current trends in computational modeling.

**RESPONSE:** We will consider this for future research; however, it is not feasible for the present study.

6. PLOS authors have the option to publish the peer review history of their article ([what does this mean?](https://track.editorialmanager.com/CL0/https:%2F%2Fjournals.plos.org%2Fdigitalhealth%2Fs%2Feditorial-and-peer-review-process%23loc-peer-review-history/1/010f0198a310556b-12f00c6d-06c9-4a2f-a53e-1f38dbabb2a3-000000/Zt0NycsIldJlVC3nbvUTEQjkmVz1RZTAcjMJwH8wU5k=224)). If published, this will include your full peer review and any attached files.


**Do you want your identity to be public for this peer review?** If you choose “no”, your identity will remain anonymous but your review may still be made public.


For information about this choice, including consent withdrawal, please see our [Privacy Policy](https://track.editorialmanager.com/CL0/https:%2F%2Fwww.plos.org%2Fprivacy-policy/1/010f0198a310556b-12f00c6d-06c9-4a2f-a53e-1f38dbabb2a3-000000/lqfVnFynamK1z6Q9jB1WAgtWlN-P_X_JftS0kPtqGzs=224).

Reviewer #1: **Yes:** Tooba Adil

Reviewer #2: No

Reviewer #3: No
