# Supplementary material for: Examining Canadian youth’s engagement with food companies via digital media
Source: PLOS Digit Health. 2025 Dec 30;4(12):e0001167. doi: 10.1371/journal.pdig.0001167 (PMC12753059; doi:10.1371/journal.pdig.0001167)
Supplement: Attachment — Submitted filename: PLOS-Review-DigitalFood.docx [file pdig.0001167.s004.docx]

**Reviewer Comments**

**Overall Comments**

This is a well-written and relevant manuscript examining youth engagement with food companies through digital media. The topic is highly pertinent to public health and digital marketing regulation. The methods are sound, and ethical and data-sharing standards are generally well met. However, the study requires minor revision to more clearly communicate the impact that this work has and situate its contribution within the existing field of research on this topic.

**Abstract**

The abstract provides a concise overview and highlights key results, but it could better position the study with respect to its impact in the broader literature. I request the following changes:

1. First sentence glosses over key points – specifically that understanding patterns of digital food behaviours in this group is important for the potential that that these patterns have on health and learned patterns that impact life-long food behaviour
2. Remove “Nonetheless, little is known about youth’s engagement with food companies via digital media.” There is substantial evidence around digital marketing strategies and their effectiveness on children (https://gh.bmj.com/content/10/2/e014667). Alternatively, clearly define “engagement” at first mention (e.g., self-reported liking/following or app downloads) to differentiate it from passive exposure.
3. Unclear if all participants had social media (line 29-3): “20.9% reported having liked, shared, or followed food/restaurant companies on social media”
4. More clearly clarify the value of your research either at the beginning or end of the abstract. The first two and last sentences of the abstract are weak. What is the value characterizing these patterns?
5. Suggest revising the key words: “food marketing; digital media; youth; food company.” There are more effective key words, particularly for indexing, including “digital advertising;” “adolescent behaviour,” “social media marketing,” “public health”

**Introduction**

The introduction effectively establishes rationale and relevance, but a few refinements are recommended:

1. The link from sentence one to sentence two is unclear. You need to provide evidence that social media leads to unhealthy diets to make the claim in sentence two. I would argue that there are many excellent, nutrition focused social media accounts.
2. Explicitly reference a conceptual framework or prior model of “digital engagement” (e.g., Montgomery et al., 2019; Tatlow-Golden et al., 2021) to anchor construct validity.
3. Emphasize the knowledge gap more directly—specifically, that previous studies examined exposure rather than engagement behaviors.
4. Overall, the introduction is long and unnecessarily verbose. I suggest the authors improve the conciseness and clarify. For examples, authors could remove “have been shown”, which is written several times; “boys have been shown to more commonly” 🡪 “boys more commonly”

**Methods**

The methodology section is generally clear and ethically sound, with appropriate approvals and consent procedures. The following improvements will enhance rigour and reproducibility.

1. What do you mean that “recruitment was stratified by”? Were there representation targets reflective of the general population applied?
2. Is stratifying by 10-12 and 13-17 commonly done in Canada (e.g., by Statistics Canada)? If so, please provide a reference to line 186.
3. If possible, I would suggest including the analytical code as supplementary material for reproducibility.
4. Justify the decision to collapse race/ethnicity into two groups (White vs minority) given potential loss of nuance. Was this a sample size decision? Substantial space is devoted to explaining the race/ethnicity categories, yet there were not included, Perhaps more to a supplemental table.
5. Clarify how engagement measures were operationalized and whether the survey referenced examples or verified responses.
6. Describe how missing responses were handled (e.g., listwise deletion) and quantify excluded cases, particularly for the statistical analyses.
7. Indicate whether weights or post-stratification adjustments were used to improve representativeness of the population included.
8. Please included details on any statistical model checks (e.g., multicollinearity or goodness-of-fit statistics) done to ensure validity of results.
9. What reporting guidelines were used?

**Results**

The results are comprehensive and logically structured, with clear tables and appropriate reporting of odds ratios and confidence intervals. However, additional detail would strengthen clarity and analytical transparency. I am recommending the following suggested revisions:

1. Participant Flow: Include a brief flow diagram (or numerical description) showing recruitment, exclusions, and final analytic sample.
2. Report response rate in paragraph 1.
3. Re: “Liking, sharing, or following food and restaurant companies on social media”: It is unclear if all included individuals have social media
4. Consider adding a visual summary figure (e.g., a forest plot or grouped bar chart of odds ratios).

**Discussion**

The discussion is insightful and well-organized, successfully linking findings to existing research and policy implications. I have no further revisions to suggest.
