# Supplementary material for: Examining Canadian youth’s engagement with food companies via digital media
Source: PLOS Digit Health. 2025 Dec 30;4(12):e0001167. doi: 10.1371/journal.pdig.0001167 (PMC12753059; doi:10.1371/journal.pdig.0001167)
Supplement: Attachment — Submitted filename: PLOS-Review-DigitalFood_Nov 19.docx [file pdig.0001167.s005.docx]

**Manuscript ID:** PDIG-D-25-00443

**Title:** Examining Canadian youth’s engagement with food companies via digital media

**Authors:** Laura Vergeer, Meghan Pritchard, Carolina Soto, Elise Pauzé, Ashley Amson, Dana Lee Olstad, Monique Potvin Kent

**NOTE:** The page and line numbers below refer to the manuscript version with Track Changes.

**Reviewer Comments**

**Overall Comments**

This is a well-written and relevant manuscript examining youth engagement with food companies through digital media. The topic is highly pertinent to public health and digital marketing regulation. The methods are sound, and ethical and data-sharing standards are generally well met. However, the study requires minor revision to more clearly communicate the impact that this work has and situate its contribution within the existing field of research on this topic.

We thank the reviewer for their feedback on our manuscript. We have addressed their comments in our responses below and in the revised manuscript.

**Abstract**

The abstract provides a concise overview and highlights key results, but it could better position the study with respect to its impact in the broader literature. I request the following changes:

1. First sentence glosses over key points – specifically that understanding patterns of digital food behaviours in this group is important for the potential that that these patterns have on health and learned patterns that impact life-long food behaviour

In the first sentence, we have added that digital food marketing to youth is concerning, in part, because of its potential to shape lifelong food behaviours (page 2, line 20).

2. Remove “Nonetheless, little is known about youth’s engagement with food companies via digital media.” There is substantial evidence around digital marketing strategies and their effectiveness on children (https://gh.bmj.com/content/10/2/e014667). Alternatively, clearly define “engagement” at first mention (e.g., self-reported liking/following or app downloads) to differentiate it from passive exposure.

We agree that there has been substantial evidence around digital marketing strategies and their effectiveness, but the focus of this study is on engagement (liking/sharing/following, app downloads). There have been relatively few studies examining youth’s engagement with food companies via digital media. The study referenced by the reviewer is a scoping review of the impact of the social media industry as a commercial determinant of health on the digital food environment for youth. It does not reference engagement via digital media, or liking/sharing/commenting/following food companies on social media. We have, however, added “particularly in Canada” to this sentence given that while there are a few studies globally that have examined youth’s digital engagement with food companies, there have has been no Canadian research on this topic (page 2, line 23). We have also added a definition of “engagement” upon first use, as suggested by the reviewer (Abstract, page 2, lines 21-22).

3. Unclear if all participants had social media (line 29-3): “20.9% reported having liked, shared, or followed food/restaurant companies on social media”

This question did not ask specifically about whether participants had social media. Presumably, the 20.9% did have social media if they were reporting having liked/shared/followed food companies. Among the total sample, there may have been some participants who did not have social media and therefore responded “no” to this question; we have added this as a study limitation (Discussion section, page 25, lines 565-566).

4. More clearly clarify the value of your research either at the beginning or end of the abstract. The first two and last sentences of the abstract are weak. What is the value characterizing these patterns?

We have edited the first and last sentences of the abstract (pages 2-3, lines 19-20, 55-57).

5. Suggest revising the key words: “food marketing; digital media; youth; food company.” There are more effective key words, particularly for indexing, including “digital advertising;” “adolescent behaviour,” “social media marketing,” “public health”

We have added the key words suggested by the reviewer (page 3, lines 59-60).

**Introduction**

The introduction effectively establishes rationale and relevance, but a few refinements are recommended:

1. The link from sentence one to sentence two is unclear. You need to provide evidence that social media leads to unhealthy diets to make the claim in sentence two. I would argue that there are many excellent, nutrition focused social media accounts.

We have added this to the first sentence of the second paragraph (page 5, line 98).

2. Explicitly reference a conceptual framework or prior model of “digital engagement” (e.g., Montgomery et al., 2019; Tatlow-Golden et al., 2021) to anchor construct validity.

We have added an article about the ‘food and beverage cues in digital marketing model’ (reference #19, page 6, lines 130-133).

3. Emphasize the knowledge gap more directly—specifically, that previous studies examined exposure rather than engagement behaviors.

As cited in the Introduction, some studies have examined engagement behaviours (e.g., references #9, 18, 22). We therefore feel the description of the knowledge gap is accurate as is.

4. Overall, the introduction is long and unnecessarily verbose. I suggest the authors improve the conciseness and clarify. For examples, authors could remove “have been shown”, which is written several times; “boys have been shown to more commonly” 🡪 “boys more commonly”

We have removed instances of “have been shown” and similar text to make the introduction slightly more concise.

**Methods**

The methodology section is generally clear and ethically sound, with appropriate approvals and consent procedures. The following improvements will enhance rigour and reproducibility.

1. What do you mean that “recruitment was stratified by”? Were there representation targets reflective of the general population applied?

These data were collected as part of a survey that aimed to examine differences in youth’s self-reported digital food marketing exposure by gender. We therefore aimed to recruit equal numbers of youth from Ontario and Quebec (due to their differing food marketing policy environments), equal numbers of children and adolescents (since most marketing policies, including Quebec’s, apply to children <13 years-old), and equal numbers of males and females (since we were interested in examining gender differences). As acknowledged in our study limitations section (page 25, lines 555-558), our sample cannot be considered representative and generalizability is limited.

2. Is stratifying by 10-12 and 13-17 commonly done in Canada (e.g., by Statistics Canada)? If so, please provide a reference to line 186.

Stratifying by children (aged 12 years and under) and adolescents (aged 13-17 years) is commonly done in food marketing research because many government restrictions and voluntary food industry policies concerning food marketing only apply to children under 13 years of age. Notably, Quebec’s Consumer Protection Act only applies to children aged 13 and under. That is why we stratified by this age group. This is explained in a previously published article by our group based on this survey data; we added a reference to this article in the Methods section (reference #27, page 8, line 186).

3. If possible, I would suggest including the analytical code as supplementary material for reproducibility.

Given that most of our analysis were descriptive, we do not feel this is necessary as these analyses can easily be reproduced without code. The logistic regression analyses were also relatively basic and easily reproducible.

4. Justify the decision to collapse race/ethnicity into two groups (White vs minority) given potential loss of nuance. Was this a sample size decision? Substantial space is devoted to explaining the race/ethnicity categories, yet there were not included, Perhaps more to a supplemental table.

Race/ethnicity was collapsed into two categories due to relatively small numbers of observations in the racial/ethnic minority categories, given that approximately 75% of the sample identified as White. We have added this explanation to the methods section (page 10, lines 223-224). We also added a breakdown of the race/ethnicity categories as Supplementary Material (Supplementary Table 1).

5. Clarify how engagement measures were operationalized and whether the survey referenced examples or verified responses.

The methods section describes the questions exactly as they were asked to participants, including any examples (page 10-11, lines 231-250).

Quality control measures for the survey are described on page 9, lines 198-201.

6. Describe how missing responses were handled (e.g., listwise deletion) and quantify excluded cases, particularly for the statistical analyses.

This is described in our “statistical analysis” section (page 11-12); we added a participant flowchart to make this clearer (Figure 1).

7. Indicate whether weights or post-stratification adjustments were used to improve representativeness of the population included.

Weights or post-stratification adjustments were not used and so this is not mentioned in the manuscript.

8. Please included details on any statistical model checks (e.g., multicollinearity or goodness-of-fit statistics) done to ensure validity of results.

We have added this to the methods section (page 13, lines 282-283).

9. What reporting guidelines were used?

We followed the STROBE reporting guideline for cross-sectional studies. We have now specified this in the Study Design section (page 8, lines 182-183).

**Results**

The results are comprehensive and logically structured, with clear tables and appropriate reporting of odds ratios and confidence intervals. However, additional detail would strengthen clarity and analytical transparency. I am recommending the following suggested revisions:

1. Participant Flow: Include a brief flow diagram (or numerical description) showing recruitment, exclusions, and final analytic sample.

We have added a flow diagram (Figure 1, page 39).

2. Report response rate in paragraph 1.

The survey was administered and managed by Leger Marketing and Quest Mindshare. We therefore do not have information about the response rate.

3. Re: “Liking, sharing, or following food and restaurant companies on social media”: It is unclear if all included individuals have social media

This question did not ask specifically about whether participants had social media. Presumably, the 20.9% did have social media if they were reporting having liked/shared/followed food companies. Among the total sample, there may have been some participants who did not have social media and therefore responded “no” to this question; we have added this as a study limitation (Discussion section, page 25, lines 565-566).

4. Consider adding a visual summary figure (e.g., a forest plot or grouped bar chart of odds ratios).

We feel this information is presented clearly and comprehensively as is in Table 2. Adding a forest plot or grouped bar chart of odds ratios would be duplicative.

**Discussion**

The discussion is insightful and well-organized, successfully linking findings to existing research and policy implications. I have no further revisions to suggest.

We appreciate the positive feedback.

**Reviewers' Comments:**

Reviewer's Responses to Questions

**Comments to the Author**

1. If the authors have adequately addressed your comments raised in a previous round of review and you feel that this manuscript is now acceptable for publication, you may indicate that here to bypass the “Comments to the Author” section, enter your conflict of interest statement in the “Confidential to Editor” section, and submit your "Accept" recommendation.

Reviewer #4: (No Response)

Reviewer #5: All comments have been addressed

Reviewer #6: (No Response)

2. Does this manuscript meet PLOS Digital Health’s [publication criteria](https://track.editorialmanager.com/CL0/https:%2F%2Fjournals.plos.org%2Fdigitalhealth%2Fs%2Fjournal-information%23loc-criteria-for-publication/1/010f019a482deb90-634b774b-1cba-463e-a7c2-7bd3e8a8faa6-000000/fhiUCcj1JCU8H-SQVXZNrXp2MqgSSSeSGFpxLpAA4hc=235)? Is the manuscript technically sound, and do the data support the conclusions? The manuscript must describe methodologically and ethically rigorous research with conclusions that are appropriately drawn based on the data presented.

Reviewer #4: Yes

Reviewer #5: Yes

Reviewer #6: Yes

3. Has the statistical analysis been performed appropriately and rigorously?

Reviewer #4: Yes

Reviewer #5: Yes

Reviewer #6: Yes

4. Have the authors made all data underlying the findings in their manuscript fully available (please refer to the Data Availability Statement at the start of the manuscript PDF file)?

The [PLOS Data policy](https://track.editorialmanager.com/CL0/https:%2F%2Fjournals.plos.org%2Fdigitalhealth%2Fs%2Fdata-availability/1/010f019a482deb90-634b774b-1cba-463e-a7c2-7bd3e8a8faa6-000000/Ojomluv-RFd1jEYKGeXlRDiTNKh8_K74uWPYRv7IjaI=235) requires authors to make all data underlying the findings described in their manuscript fully available without restriction, with rare exception. The data should be provided as part of the manuscript or its supporting information, or deposited to a public repository. For example, in addition to summary statistics, the data points behind means, medians and variance measures should be available. If there are restrictions on publicly sharing data—e.g. participant privacy or use of data from a third party—those must be specified.

Reviewer #4: Yes

Reviewer #5: Yes

Reviewer #6: Yes

5. Is the manuscript presented in an intelligible fashion and written in standard English?

PLOS Digital Health does not copyedit accepted manuscripts, so the language in submitted articles must be clear, correct, and unambiguous. Any typographical or grammatical errors should be corrected at revision, so please note any specific errors here.

Reviewer #4: Yes

Reviewer #5: Yes

Reviewer #6: Yes

6. Review Comments to the Author

Please use the space provided to explain your answers to the questions above. You may also include additional comments for the author, including concerns about dual publication, research ethics, or publication ethics. (Please upload your review as an attachment if it exceeds 20,000 characters)

Reviewer #4: This study shows a sound methodology, and the contribution is important. To note the comments and responses from the previous reviewers:

1. A lack of an outcome of unhealthy eating. The discussion of studies that note a relationship between marketing exposure and unhealthy eating support the outcome, but it does reduce the inferences that this study can make.
2. Regarding previous publications on this dataset. The authors distinguish between previous research about “exposure” to digital marketing and the current study exploring “engagement” with digital marketing. The current analysis is limited engagement specifically.
3. The choice to undertake logistic regression only limits the analysis’ ability to explore the factors implementing the outcome. But I accept the lack of feasibility to address this in the current study and note the points addressed in the discussion.

The authors have responded to reviewers’ comments adequately and provide discussion of the limitations. The accepted limitations restrict the contribution of this research to a board association between some sociodemographic factors and digital food marketing engagement, with difficulty to extrapolate much further. However, there is a need for contributions in this area and the research feeds into a desire for policy intervention. The translational potential suggests this contribution is important and worthwhile, despite the limitations.

An additional note is that more focus should be applied to future qualitative research as a discussion point. If a key outcome of this research is to result in policy development and implementation, a better understand of “why” certain groups are more likely to engage in digital food marketing, and consumer input into policy development, is required for effective policy. Qualitative research is a good option, and recommendations should involve the inclusion of consumers in future policy development.

We thank the reviewer for their positive feedback on our manuscript. We have added a sentence to the Discussion about the need for qualitative research and consumer input into policy development (page 24, lines 544-546).

Reviewer #5: The authors have addressed all prior points thoroughly. I have no further comments at this stage. Congratulations on the careful and well-executed manuscript.

We thank the reviewer for their positive comments on our manuscript.

Reviewer #6: Excellent work. Please see minor revisions attached.

Thanks to the reviewer for their feedback. We have addressed their comments in our responses to reviewers and in the revised manuscript.

7. PLOS authors have the option to publish the peer review history of their article ([what does this mean?](https://track.editorialmanager.com/CL0/https:%2F%2Fjournals.plos.org%2Fdigitalhealth%2Fs%2Feditorial-and-peer-review-process%23loc-peer-review-history/1/010f019a482deb90-634b774b-1cba-463e-a7c2-7bd3e8a8faa6-000000/wjvECixzmLxOH2Om2f2HBHAW3jpFeBmbG4TCx5NNhzg=235)). If published, this will include your full peer review and any attached files.


**Do you want your identity to be public for this peer review?** If you choose “no”, your identity will remain anonymous but your review may still be made public.


For information about this choice, including consent withdrawal, please see our [Privacy Policy](https://track.editorialmanager.com/CL0/https:%2F%2Fwww.plos.org%2Fprivacy-policy/1/010f019a482deb90-634b774b-1cba-463e-a7c2-7bd3e8a8faa6-000000/Z6FzEsNTmYTy30F6P3EmY_kzQPMBYY381ZCICscGuJE=235).

Reviewer #4: **Yes:** Calum Nicholson

Reviewer #5: **Yes:** Shruti Muralidharan

Reviewer #6: No

[NOTE: If reviewer comments were submitted as an attachment file, they will be attached to this email and accessible via the submission site. Please log into your account, locate the manuscript record, and check for the action link "View Attachments". If this link does not appear, there are no attachment files.]
